# Reproductive Decision Support: Preferences and Needs of Couples at Risk for Hereditary Cancer and Clinical Geneticists

**DOI:** 10.1007/s10897-017-0204-6

**Published:** 2018-01-25

**Authors:** Kelly Reumkens, A. J. G. van Oudheusden, J. J. G. Gietel-Habets, M. H. E. Tummers, C. E. M. de Die-Smulders, L. A. D. M. van Osch

**Affiliations:** 1 Department of Clinical Genetics, Maastricht University Medical Centre+, Maastricht, the Netherlands; 2 GROW School for Oncology and Developmental Biology, Maastricht University Medical Centre+, Maastricht, the Netherlands; 3 Department of Health Promotion, Maastricht University Medical Centre+, Maastricht, the Netherlands; 4 School CAPHRI, Care and Public Health Research Institute, Maastricht University Medical Centre+, Maastricht, the Netherlands

**Keywords:** Oncology, Hereditary cancer, Counseling, Child wish, Informed decision-making, Decision aid, Prenatal diagnosis and preimplantation genetic diagnosis

## Abstract

For couples at high risk of transmitting a cancer predisposition to offspring, reproductive decision-making can be challenging. As the choice between available reproductive options is preference-sensitive, the use of a decision aid can support these couples in their decisional process. The present study aims to investigate preferences and needs of involved stakeholders regarding the development and implementation of a patient decision aid. Semi-structured interviews assessing the needs and preferences regarding the content and functionalities of a decision support program were conducted among seven couples at risk for hereditary cancer and among eight clinical geneticists involved in oncogenetic counseling. Many similarities were found between the expressed preferences and needs of both stakeholder groups concerning the content, barriers and facilitating factors regarding the use of the decision aid, and its implementation. Emphasis was placed on the use of simple non-medical language, an extensive explanation of the procedures and techniques used in prenatal diagnosis (PND) and preimplantation genetic diagnosis (PGD), and the role of health care providers to refer couples to the decision aid. Both stakeholder groups were in favor of incorporating narrative stories in the decision aid. Integrating the present findings with knowledge on reproductive decisional motives and considerations is essential in guiding the development of a decision aid that corresponds to the preferences and needs of end-users. Trial registration: NTR5467

## Background

Individuals with a family history of cancer may face the decision on whether or not to undergo genetic testing. This decision may set in motion a cascade of decisions, including deciding on whether or not to inform family members and, if available, whether or not to take up periodic screening and preventive therapies in case of a confirmed mutation. Carriers of reproductive age additionally face challenging decisions regarding one’s wishes to have children and the welfare of their future child(ren) (Dekeuwer and Bateman 2013; Derks-Smeets et al. 2014). As most types of hereditary cancer are transmitted in an autosomal dominant pattern, there is a 50% risk of transmitting the mutation. This knowledge plays a substantial role in the reproductive decision-making process (Niermeijer et al. 2008). Carrier status impacts the decision of mutation carriers to have biological children (Chan et al. 2016). Apart from deciding to not have children, couples may decide to pursue options to have non-biological children (e.g. adoption, foster parenting). Most couples, however, pursue their wish to have biological child(ren) (Chan et al. 2016).

Carrier couples who want a biological child can opt for natural conception without genetic testing and accept the risk of passing on the susceptibility to cancer to the child, prenatal diagnosis (PND) assuming the intention to terminate the pregnancy (TOP) in case the fetus is a carrier of the genetic mutation (de Die-Smulders et al. 2013) or preimplantation genetic diagnosis (PGD). PGD involves a multi-stage diagnostic process in which embryos derived by in vitro fertilization (IVF) are screened for the presence of the familial mutation before pregnancy is established. Subsequently, only embryos without the mutation are transferred into the uterus (de Die-Smulders et al. 2013). Previous research showed that approximately half of couples consider PND or PGD after receiving a positive genetic test result for hereditary cancer (Fortuny et al. 2009) and the majority think PND and PGD should be offered to mutation carriers (Chan et al. 2016).

In deliberating the options for fulfilling one’s wish to have children, couples carefully consider various personal values and advantages and disadvantages of all reproductive options, previously categorized into physical, psychological, social, ethical, and practical considerations (Derks-Smeets et al. 2014). Research has demonstrated that couples often experience the reproductive decision-making process as very difficult (Dekeuwer and Bateman 2013; Dommering et al. 2010; Ormondroyd et al. 2012). Feelings of uncertainty, regret, and guilt are common (Derks-Smeets et al. 2014). In addition to reproductive counseling, decision support may be helpful to support couples during reproductive decision-making (Derks-Smeets et al. 2014; Ormondroyd et al. 2012; Quinn et al. 2010a, 2012). Although recent studies have provided more insight into the reproductive decision-making process of carrier couples (Dekeuwer and Bateman 2013; Derks-Smeets et al. 2014; Dommering et al. 2010; Ormondroyd et al. 2012), and the application of decision support has been advocated (Derks-Smeets et al. 2014; Quinn et al. 2010a, b), currently, no structural decision support is available. High-quality evidence shows positive effects of decision aids on various patient outcomes, such as increased knowledge regarding potential options, reduced decisional conflict, and facilitation of informed and value-based decision-making (Juraskova et al. 2014; O’Connor and Jacobsen 2003; Stacey et al. 2011).

The present study is part of a larger research project on the development and implementation of an online patient decision aid. The decision aid is developed according to the International Patient Decision Aids Standards (IPDAS). The first step in the development process is to provide insight into the preferences and needs of important stakeholders regarding the content and implementation of the decision aid. Both patients’ and practitioners’ decisional needs may influence the quality of the decision and a thorough understanding of the needs of both stakeholder groups is essential to ensure successful development and promotion of the use of the intended decision aid (Coulter et al. 2012, 2013; Jacobsen et al. 2013). In this manuscript, we present the outcomes of a needs assessment among both groups as the first step towards the development of a patient decision aid for reproductive decision-making among couples at risk for hereditary cancer.

## Methods

Semi-structured interviews were conducted among couples at risk for hereditary cancer (Study 1) and clinical geneticists (Study 2).

### Participants

The Clinical Genetics Department of the Maastricht University Medical Centre (MUMC+) is the only department in the Netherlands authorized to perform PGD. The MUMC+ has set up a database of couples who have had reproductive counseling for hereditary cancer since 2008 when PGD was approved for late-onset inherited cancer predisposition syndromes in the Netherlands. Seventeen couples from this database who have had reproductive counseling for hereditary cancer between January 2013 and January 2015 were randomly selected and contacted to participate in Study 1. Couples were eligible for participation if one partner was a mutation carrier for hereditary cancer for which PND and PGD are available in the Netherlands, if both partners were 18 years or older, and if both partners had sufficient knowledge of the Dutch language. Couples received an invitational letter for participation, an informative letter, and an informed consent form for each partner. For Study 2, clinical geneticists of the nine Clinical Genetics Departments in the Netherlands who were involved in oncogenetic counseling were invited by e-mail to participate with exclusion of the clinical geneticists of the MUMC+ who are all directly involved in the project.

### Instrumentation and Procedures

Separate semi-structured topic guides were developed for guidance of the dyadic and individual interviews. The content of both topic guides was focused on the content, layout, format, dissemination, and implementation strategies of the patient decision aid (see Table 1). Clinical geneticists received additional questions concerning their professional perspectives regarding the development and implementation of the decision aid in order to facilitate structural decision support use and referral within consultations. The interviews were conducted by two researchers (K.R. and A.O.) and held in the home environment of couples with both partners participating and at convenient workplaces for clinical geneticists. All interviews were audiotaped. Participants were asked to fill out a brief questionnaire prior to the start of the interview. Apart from demographic factors (e.g., age and gender) couples were asked about their carrier status (e.g., type of hereditary cancer syndrome), current reproductive preferences (e.g., natural conception without genetic testing, PND, PGD, and refraining from fulfilling one’s wish to have a child(ren)), internet experience (1 = no experience, 4 = a lot of experience), and expectations concerning the expected use of a decision aid (1 = definitely not, 5 = definitely). Clinical geneticists were also asked about work experience in the counseling of couples at risk for hereditary cancer (1 = less than 1 year of experience, 5 = more than 10 years of experience) and experience with decision aids (1 = no experience, 4 = a lot of experience).

**Table 1:** Overview of main questions asked during dyadic (Study 1) and individual (Study 2) interviews

Study 1. Couples
Content
• What information do you consider important in order to make an appropriate reproductive decision?
• What kind of information assisted or could have assisted you in making a reproductive decision?
• Please specify your preferences with respect to functionalities and/or applications that can be included in the decision aid.
Layout
• What do you think a decision aid should look like (appearance)?
• What are your wishes regarding the layout of the decision aid?
Barriers and facilitating factors
• Please identify potential barriers for yourself regarding the use of the decision aid.
• Which suggestions do you have in order to prevent these barriers?
• Please identify facilitating factors for yourself regarding the use of the decision aid.
Dissemination and implementation
• How and when would you like to be informed about the decision aid?
• When and where would you have preferred to use the decision aid?
Study 2. Clinical geneticists
Content
• What information do you consider important for couples in order to make an informed decision?
• What are your ideas with regard to the inclusion of functionalities and/or applications in the decision aid?
Layout
• What do you think a decision aid should look like (appearance)?
• What are your wishes regarding the layout of the decision aid?
Barriers and facilitating factors
• What do you consider potential barriers for couples to use the decision aid?
• What do you consider potential facilitating factors for couples to use the decision aid?
• What would be barriers for yourself to refer to the decision aid as intended?
• What would be facilitating factors for yourself to refer to the decision aid as intended?
Dissemination and implementation
• What are your preferences with respect to the availability of the decision aid?
• What do you consider the best point in time to use the decision aid?
• How do you consider your role as clinical geneticist with regard to the implementation of the decision aid?

### Data Analysis

Qualitative data derived from the audiotaped interviews were transcribed verbatim and independently analyzed by two researchers (K.R. and A.O.). A phenomenological investigation method was used to explore preferences and needs of couples at risk for hereditary cancer and clinical geneticists (Husserl 1964). Open and axial coding was performed to derive and categorize main themes. Coding of the data was done by two independent researchers and comparison of coding was conducted in order to reach consensus. Data from the brief questionnaires were analyzed by descriptive statistics using SPSS version 23.

## Results

Fifteen semi-structured interviews with seven couples (*n* = 14 individuals) and eight clinical geneticists were conducted between April and June 2015 with an average duration of 62 minutes (range 40–75). After five couples and six clinical geneticists, data saturation seemed to be achieved. Two additional interviews were conducted, in which no new or salient data were generated and data collection was concluded.

### Study 1: Needs Assessment Among Couples

#### Couples’ Characteristics

Seven couples gave informed consent for participation (response rate 41.2%) with a mean age of 33.4 years for males (SD = 3.0) and 30.6 years (SD = 2.8) for females. Table 2 shows an overview of couples’ characteristics. Main reasons for non-participation were a lack of time and not wanting to relive the psychological burden associated with reproductive decision-making. The majority (79%) expressed a positive intention towards the use of a decision aid, if it had been available at the time of their reproductive decision (mean = 4.29, SD = 0.99). Most respondents had ample experience with internet and computers (93%; mean = 3.71, SD = 0.61).

**Table 2:** Couples’ characteristics

Characteristic	*n*	Percentage (%)
Gender		
Male Female	7 7	50.0 50.0
Mean age (in years)		
Male Female	30.6 (SD = 2.8) 33.4 (SD = 3.0)	
Education		
Low Middle High	1 5 8	7.1 35.8 57.1
Gender of carriers		
Female	7	100.0
Mutation type		
BRCA 1/2 Lynch Familial adenomatous polyposis Retinoblastoma Paraganglioma Hereditary diffuse gastric cancer	2 1 1 1 1 1	28.6 14.3 14.3 14.3 14.3 14.3
Reproductive decision		
PGD Natural conception	10 4	71.4 28.6

Seven couples (*n* = 14 individuals) participated in the interviews

#### Preferences and Needs Regarding the Content of the Decision Aid

In addition to the main reproductive options (natural conception without genetic testing, PND, and PGD), the principles of decision support were explained prior to the interviews.

##### Informational Content of the Decision Aid and Presentation of Information

Couples expressed a need for a complete explanation in the decision aid of the procedures and techniques used in PND and PGD, including procedures for IVF and pregnancy termination. Participants put particular emphasis on the duration, physical consequences, and the expected psychological burden of the PND and PGD trajectory.

For someone who is not specialized in genetics, a clear and comprehensive overview of the medical process provided in the decision aid gives you an understanding of the complexity, which enables you to understand the required time and therefore be more patient. [C7]

Also, information about success rates, such as the chance of pregnancy with PGD and risks, such as the likelihood of a miscarriage after PND, were mentioned by the majority as having a significant influence on reproductive decision-making. Some couples added that duration of the procedures and family planning are strongly related and should therefore also be emphasized in the decision aid.

Family planning might be different than expected. If you opt for PGD, a large family is less realistic. So, family planning should be part of the decision aid. [C6]

One couple pointed out that clear information should be provided about the time and effort required to prepare for PGD (e.g., visitations required with various health care providers). Another couple pointed out that it would be helpful to explain why family members need to be involved in case one opts for PGD and how the involvement of family members can be related to a longer duration of the trajectory. The majority emphasized the need for simple non-medical language and comprehensible content, as couples would like to share the information in the decision aid with relatives who are generally unfamiliar with the subject.

##### Functionalities and Applications in the Decision Aid

Most couples were of the opinion that images may contribute to the creation of a realistic impression regarding the procedures of PND and PGD. However, some also expressed their concern regarding the complexity of images. Additionally, the majority recognized the use of videos as helpful in demonstrating procedures and techniques. None of the couples thought that it would be helpful to present some type of a conclusion or advice regarding a “best fitting option” after completing the decision aid. Instead, most couples preferred some form of an evaluation, such as an overview of couples’ preferences and values. The majority was of the opinion that it would be better to provide an overview and let couples interpret this overview by themselves. A few couples acknowledged that the inclusion of a chat application or discussion forum could be valuable. However, a regularly expressed concern was the risk of receiving incorrect information.

Reliable and tailored information is necessary. A forum may raise unnecessary concerns as certain issues may not be applicable to all couples. [C7]

A potential alternative indicated by almost all couples was the provision of narrative stories (i.e., personal stories of couples who have already made a reproductive decision). Reading stories of experienced couples would make the decision-making process more personal as couples do not only want to read about scientific facts. One couple added it would be helpful to know that there are more couples who are struggling with the same problems.

##### Barriers and Facilitating Factors Regarding the Use of the Decision Aid

Several couples indicated the use of difficult language (e.g., medical abbreviations) and an extensive amount of text as potential barriers to the use of the decision aid. Although a long duration was not considered as a barrier by most couples, a maximum duration of 60 minutes was recommended. In order to promote first use of the decision aid, the majority indicated that reliability of the information and expertise of the development team were important facilitating factors. Furthermore, all couples were of the opinion that referral to the decision aid by their health care provider would encourage use of the decision aid.

##### Implementation of the Decision Aid

All but one couple agreed that the best time for implementing the decision aid would be in between the moment of receiving a positive genetic test result and the follow-up consultation at one of the clinical genetic departments. Providing the decision aid before follow-up consultations was desirable as the decision aid may raise important questions to discuss with health care providers. Several couples believed that this may lead to a more interactive consultation. However, some concerns were expressed about providing the decision aid immediately after confirmation of a genetic mutation as this can be an emotionally challenging time. All couples agreed that it is the role of the health care provider to choose the best moment to refer couples to the decision aid. To foster implementation, most couples suggested including information about the decision aid in the standard report they receive after consultation.

### Study 2: Needs Assessment Among Clinical Geneticists

#### Clinical Geneticists’ Characteristics

All eight clinical geneticists who were invited participated in the dyadic interviews (two males and six females) with a mean age of 53.0 years (SD = 2.8) for males and 45.8 years (SD = 8.3) for females. The majority had more than 10 years of work experience in the area of oncogenetic counseling; however, half of the clinical geneticists had no experience with the use of patient decision aids (mean = 1.50, SD = 0.53).

### Preferences and Needs Regarding the Content of the Decision Aid

#### Informational Content of the Decision Aid and Presentation of Information

Although clinical geneticists agreed upon natural conception without genetic testing, PND, and PGD as main reproductive options in the decision aid, two clinical geneticists indicated that attention to other reproductive options (e.g., refraining from fulfilling one’s wish to have a child, and use of donor gametes) would also be helpful to make couples aware of the availability of these options. Furthermore, the majority considered a complete explanation of the procedures and techniques used in PND and PGD (e.g., duration, physical and emotional burden, inclusion criteria, IVF, and pregnancy termination) and information about success rates (e.g., pregnancy) as important issues to be included in the decision aid. According to the majority, the decision aid should clearly indicate the required time investment for PGD (e.g., visitations required with various health care providers) and the timing of PND procedures (i.e., required duration of pregnancy). Also, the waiting time related to the genetic test result and the possibility of a moral dilemma concerning a pregnancy termination with PND were mentioned as important issues to be included in the decision aid. Clinical geneticists agreed the decision aid should create realistic expectations and therefore also negative features (e.g., the risk of not having unaffected embryos to transfer with PGD) and risks (e.g., increased risk of miscarriage with PND) should be described.

#### Functionalities and Applications in the Decision Aid

Six clinical geneticists mentioned that the use of visual materials, especially videos, in addition to text would be valuable to create a realistic impression of procedures and techniques used in PND and PGD. Three clinical geneticists suggested the importance of balancing the language used in the decision aid to relate to people of lower and higher education. To accomplish this, all clinical geneticists agreed on the use of different presentation formats (e.g., text, videos, images, graphics). Additionally, five clinical geneticists preferred some form of an evaluation, such as an overview of couples’ preferences and values, after completing the decision aid over a conclusion or advice regarding a “best fitting option.”

It is better to list all points discussed in the decision aid together with couples’ answers, instead of providing a conclusion. Couples can interpret their answers by themselves together with a health care provider. [CG 3]

All but one of the clinical geneticists expressed concerns regarding a chat application, with the main concern that incorrect information could be presented.

Sometimes, the first part of a consultation consists of explaining inaccuracies and only after that you can start discussing facts. That is something that can also occur as a result of a chat application included in the decision aid. [CG 2]

Although objectivity and the provision of balanced information remained an essential point, most clinical geneticists indicated that the use of narrative stories could be beneficial.

A video in which couples tell their experiences with the reproductive option of their choice would be interesting. This could absolutely be of additional value to the decision aid. But make sure it shows the whole spectrum of positive and negative stories. [CG 2]

#### Barriers and Facilitating Factors Regarding the Use of the Decision Aid

Clinical geneticists agreed upon the importance of referral to the decision aid by involved health care providers to promote its initial use. The use of difficult language (e.g., medical terms) was considered to be a hindrance for couples regardless of educational level, and a maximum duration of 60 minutes to complete the decision aid was recommended. To facilitate the sustained use of the decision aid, the majority emphasized user-friendliness and the use of evidence-based and up-to-date information.

#### Preferences and Needs Concerning the Implementation of the Decision Aid

When asked about their opinion regarding the availability of the decision aid, clinical geneticists were divided on whether the decision aid should be freely available (e.g., free access on the internet) or whether access should be restricted to eligible couples (e.g., by means of unique login data, distributed by health care providers). The preference for a freely available tool was mainly based on the idea that a larger group of potential couples could be reached, whereas those in favor of restricted access expressed concerns about reaching a wrong audience (e.g., carriers for which PND or PGD is not available).

Optimal timing for implementing the decision aid was considered to be in between the moment of receiving a positive genetic test result and follow-up consultations (e.g., aftercare and consultations regarding available reproductive options). All clinical geneticists agreed that it is the responsibility of counselors to refer to the decision aid. To promote implementation, clinical geneticists agreed that referral should not take too much effort or greatly deviate from their daily practice. Including a link to the online decision aid in the standard report counselees receive after consultation was therefore suggested by all clinical geneticists.

## Discussion

This study provides insights into the preferences and needs of couples at risk for hereditary cancer and clinical geneticists involved in oncogenetic counseling with respect to the development and implementation of a patient decision aid regarding reproductive decision-making. Couples and clinical geneticists expressed similar ideas and opinions regarding the content, barriers and facilitating factors regarding the use of the decision aid, and its implementation. Both stakeholder groups agreed on the inclusion of information about success rates, risks, procedures, and techniques used in PND and PGD and the responsibility of health care providers to refer to the decision aid in order to optimize utilization. Furthermore, the use of visual materials, especially videos, was considered important in order to create a realistic impression of procedures and techniques used in PND and PGD. Emphasis was placed on the use of simple non-medical language. Overall, there appears to be a strong preference among both stakeholder groups for incorporating narrative stories that detail the experiences of couples with reproductive decision-making.

### Research Recommendations

Currently, insufficient evidence exists about the effectiveness of narrative stories on informed decision-making and how to incorporate these stories in decision aids (Bekker et al. 2012, 2013). Future research is therefore necessary to explore essential elements for the content of narrative stories and its effectiveness on decision-making.

### Study Limitations

A limitation of Study 1 relates to the fact that only couples who had already made a reproductive decision were included. These couples had to reflect in retrospect on their reproductive decision-making process, which may have led to recall bias. These couples, however, have extensive experience with the decision-making process and may therefore be better able to evaluate and describe their needs and wishes throughout the entire process. Furthermore, only clinical geneticists were included in Study 2. Although they are likely to be most involved in the implementation and use of the decision aid, other health care providers such as genetic counselors, social workers, PGD/IVF physicians, and gynecologists may add other valuable insights.

### Practical Implications

The findings from this study, combined with results from preliminary investigations regarding reproductive decisional motives and considerations among the target group (Derks-Smeets et al. 2014), will guide the development of a patient decision aid on reproductive options for couples at risk for hereditary cancer and child wish. Ultimately, it is expected that this decision support will enable end-users to make an informed decision, which may lessen the negative psychological impact of decision-making on couples’ daily life and well-being.

## Conclusion

Although the reproductive decision-making process of couples with hereditary cancer has increasingly been investigated and the provision of decision support is suggested, currently, no specific decision support tool is available for this target group. The present study provides an overview of the preferences and needs of couples and clinical geneticists regarding reproductive decision support. Integrating these findings with findings regarding reproductive decisional motives and considerations from previous studies is essential in guiding the development of a patient decision aid that optimally corresponds to the preferences and needs of end-users.
